# Schizophrenia and bipolar disorder: a comparative analysis of genetic and brain network connectivity

**DOI:** 10.1017/S0033291726104413

**Published:** 2026-06-19

**Authors:** Hongyan Ren, Yunjia Liu, Yunqi Huang, Yiguo Tang, Liling Xiao, Yulu Wu, Siyi Liu, Yubing Yin, Qianshu Ma, Minhan Dai, Shiwan Tao, Min Xie, Mingli Li, Tao Li, Qiang Wang

**Affiliations:** 1 Mental Health Center & National Center for Mental Disorders, West China Hospital of Sichuan University, Chengdu, Sichuan, China; 2 Aﬃliated Mental Health Center Hangzhou Seventh People’s Hospital, Zhejiang University School of Medicine, Hangzhou, China; 3 Liangzhu Laboratory, MOE Frontier Science Center for Brain Science and Brain-machine Integration, State Key Laboratory of Brain-machine Intelligence, Zhejiang University, 1369 West Wenyi Road, Hangzhou 311121, China; 4 NHC and CAMS Key Laboratory of Medical Neurobiology, Zhejiang University, Hangzhou 310058, China; 5 Nanhu Brain-computer Interface Institute, Hangzhou 311100, China

**Keywords:** bipolar disorder, brain network connectivity, imaging genetics, mendelian randomization, schizophrenia, default mode network, resting-state networks, Genome-wide association study (GWAS), Genetic correlation, Limbic network, Functional connectivity, Psychiatric comorbidity

## Abstract

**Background:**

Schizophrenia (SCZ) and bipolar disorder (BD) are severe psychiatric conditions with overlapping clinical presentations, genetic risk factors, and brain network dysfunction. Whether alterations in large-scale intrinsic brain networks reflect shared or disorder-specific genetic influences remains poorly understood. Clarifying this distinction is essential for refining etiological models and improving diagnostic precision.

**Methods:**

Genome-wide inferred statistics (GWIS) were applied to decompose the genetic architecture of SCZ and BD into shared and unique components. Using resting-state network (RSN) data from the UK Biobank, functional connectivity (FC) and structural connectivity (SC) were extracted as neuroimaging phenotypes. Causal inference approaches were subsequently employed to infer potential directional relationships between brain network connectivity and each disorder.

**Results:**

Analyses revealed both common and distinct patterns of brain network connectivity associated with SCZ and BD. Notably, SC within the default mode network (DMN) exhibited opposing effects across the two disorders, suggesting divergent structural underpinnings despite clinical overlap. Additionally, SC within the limbic network (LN) and frontotemporal control network demonstrated potential causal relationships with both conditions, implicating these circuits astransdiagnostic neural substrates.

**Conclusion:**

These findings illuminate the shared and disorder-specific genetic and neural architecture underlying SCZ and BD. Integrating genome-wide genetic methods with large-scale neuroimaging data offers a powerful framework for disentangling psychiatric comorbidity and may inform more targeted diagnostic criteria and individualized treatment strategies.

## Introduction

Bipolar disorder (BD) and schizophrenia (SCZ) are debilitating psychiatric disorders with a substantial global burden (Castelpietra et al., 2022). BD manifests through alternating manic/hypomanic and major depressive episodes, while SCZ is characterized by delusions, hallucinations, disorganized thinking (speech), grossly disorganized or abnormal motor behavior, and negative symptoms (American Psychiatric Association, 2022). BD and SCZ were referred to as manic-depressive psychosis and dementia praecox, respectively, according to the Kraepelinian dichotomy (Craddock & Owen, 2005). Over a century, they are still two distinct disorders in current clinical practice, based on the Diagnostic and Statistical Manual of Mental Disorders (fifth edition text revision, DSM-5 TR) (American Psychiatric Association, 2022) and the Classification of Mental and Behavioral Disorders from International Classification of Diseases 11th revision (ICD-11). However, there is an ongoing debate between dichotomy and continuum (Cheniaux et al., 2008; Winokur, Monahan, Coryell, & Zimmerman, 1996). BD and SCZ truly share certain risk factors, clinical symptoms, and antipsychotic treatments from an epidemiological and clinical perspective (Pearlson, 2015). Furthermore, genetic studies have revealed high heritability and overlapping genetic susceptibility between BD and SCZ (Bipolar Disorder and Schizophrenia Working Group of the Psychiatric Genomics Consortium, Electronic address, 2018; Craddock, O’Donovan, & Owen, 2005), emphasizing the need to explore their genetic relationship concerning the underlying pathophysiology in the brain.

Brain network connectivity provides an integrated summary of the large-scale circuit organization, making it a suitable intermediate phenotype for imaging-genetic analyses. Brain magnetic resonance imaging (MRI) studies have provided abundant evidence that abnormal functional connectivity (FC) and structural connectivity (SC) were found and related to cognitive function and clinical syndromes in patients diagnosed with BD and SCZ (Liang et al., 2022; Qiu et al., 2021). For example, aberrant FC in anterior cingulate cortex within default mode network (DMN) and visual network (VN) was associated with different psychosis symptoms in first-episode SCZ (Qiu et al., 2021). Unfortunately, those findings play a negligible role in clinical psychiatric diagnosis and treatment, due to lack of reproducibility and consistence (Marek et al., 2022).

Accumulating evidence suggests that genetic determinants play a significant role in shaping brain network connectivity and overlap with the genetic architecture of psychiatric disorders (Medland et al., 2022). A recent genome-wide association study (GWAS) study on FC and SC within resting-state networks (RSNs) was conducted to detect genetic architecture of within-RSN FC and SC properties (Tissink et al., 2023), advancing the understanding of the complex organization of the brain networks from a genetic viewpoint. We delve into the realm of imaging genetics, targeting a comprehensive understanding of how genetic factors contribute to brain connectivity abnormalities in BD and SCZ.

In this study, we hypothesized that SCZ and BD share part of their genetic basis but also show disorder-level differences in genetic factors alternating brain network connectivity. To test this, we first performed genome-wide inferred statistics (GWIS) to separate shared and disorder-unique genetic components and examined their associations with brain connectivity. We isolated trait-specific associations after accounting for shared genetic liability and to avoid inflated signals driven by overlapping genetic influences. This analytic framework enabled us to link genetic variation to brain connectivity patterns and their contribution to SCZ and BD.

## Methods and materials

### Data sources

In this study, we aim to dissect the genetic architecture underlying SCZ and BD and their relationship with brain network connectivity alterations. All of our following analyses are summary statistics based on Supplementary Table 1. For BD and SCZ, we used the GWAS results of BD conducted by Mullins et al. (Mullins et al., 2021) including 41,917 cases and 371,549 controls, and those of SCZ conducted by Trubetskoy et al. (Trubetskoy et al., 2022) including 76,755 cases and 243,649 controls. For brain connectivity, we used the GWAS results of 16 FC and SC within RSNs conducted by Tissink et al. (2023) in the UK Biobank participants (N = 24,336). FC and SC were quantified as the average Pearson’s correlation coefficient and average fractional anisotropy of the connections within the referred seven canonical RSNs defined by Yeo et al. (2011), including the DMN, ventral attention network (VAN), dorsal attention network (DAN), visual network (VN), limbic network (LN), somatomotor network (SMN), and frontoparietal control network (FPCN). Additionally, a global measure of FC/SC was calculated for the whole-brain connections. These traits were derived using standardized parcellation and preprocessing pipelines as described in the original publication. All the above studies were carried out in the population of European ancestry.

### Genome-wide inferred statistics

**Figure 1.**
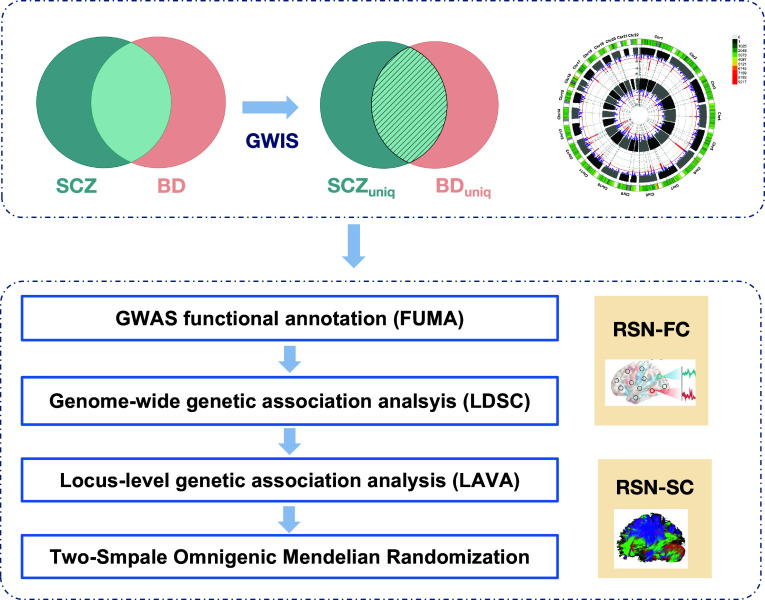
The workflow of the study. *Note*: BD; SCZ; BD_uniq_; SCZ_uniq_; RSN-FC/SC; FUMA; LDSC; LAVA; GWIS.

The overall pipeline of our study is shown in Figure 1. To detangle the relationship between SCZ and BD and identify the distinct genetic architecture for each one, we chose the GWIS (https://github.com/MichelNivard/EA_SZ/tree/ master/GWIS) (Nieuwboer et al., 2016) to derive a secondary, genetically unique GWAS result for SCZ and BD by applying the following functions to the original GWAS data:

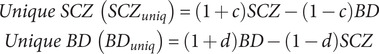

where

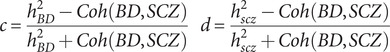




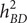
 and 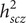 means the heritability of BD and SCZ and Coh (BD, SCZ) means coheritability between BD and SCZ.

In brief, we applied GWIS to estimate SCZ-unique (SCZ_uniq_) and BD-unique (BD_uniq_) components by removing the linear shared genetic variance between SCZ and BD, which are based on DSM-defined diagnosis. These GWIS-derived components do not imply fully independent biological pathways but rather represent disorder-associated signals once the shared portion is removed. We consistently use the terms BD, SCZ, BD_uniq_, and SCZ_uniq_ throughout the manuscript. To further understand the biological implications of two GWIS-derived components, we subsequently subjected them to the Functional Mapping and Annotation (FUMA) platform (Watanabe, Taskesen, van Bochoven, & Posthuma, 2017) and SynGO (Koopmans et al., 2019) for functional annotation and enrichment analyses.

### Global and local genetic correlation

For the first step, we chose the linkage disequilibrium-score regression (LDSC) (Bulik-Sullivan et al., 2015) to investigate the single nucleotide polymorphism (SNP)-based heritability and global genetic correlation between SCZ, BD, and 16 RSN-FC/SCs. The LDSC calculates the LD score using the 1000G EUR as the reference panel and generates a genome-wide genetic correlation (r_g_) for each trait pair. To examine genetic overlap at a finer resolution, we then applied the Local Analysis of [co]Variant Association (LAVA) (Werme, van der Sluis, Posthuma, & de Leeuw, 2022). LAVA estimates local genetic correlation within 2495 genomic regions which were predefined by Werme et al. using 1000 Genomes (EUR) data with the minimum block size set to 2500 (locations are in reference to build hg19/GRCh37) (Werme et al., 2022). For each genomic locus, LAVA generates a local genetic correlation (r_g_) for each trait pair. The nominally significant threshold was set as 0.05, and we applied false discovery rate (FDR) correction to control for multiple testing.

### Omnigenic mendelian randomization

Since the genetic correlation studies only demonstrate covariations, not a directed causal relationship between two variables, we conducted the two-sample Mendelian randomization (MR) studies to establish the causal relationship between four disorder phenotypes (both SCZ/ SCZ_uniq_ and BD/ BD_uniq_) and 16 RSN-FC/SCs. The seminal study by Boyle et al. proposed an omnigenic model for complex disorders such as BD and SCZ, in which it is postulated that in the tissues relevant to the disorder, all the expressed genes contributed, in a different role, to the pathogenesis of the disorder (Boyle, Li, & Pritchard, 2017). Based on the new disease model, omnigenic Mendelian randomization (OMR) (Wang et al., 2021) analysis provides a novel analytic framework for two-sample MR studies. The method is based on the concept of the omnigenic model, which uses genome-wide SNPs to serve as instrumental variables (IVs). OMR utilizes summary statistics from GWAS as input and relies on a composite likelihood framework for scalable computations. Each OMR analysis output includes two primary parameters: the causal effect (α) and the proportion of SNP horizontal pleiotropy effect in the outcome variable (γ). We used the R package ‘OMR’ to perform the OMR analyses between four disorder traits and 16 RSN-FC/SC traits. For each analysis, the brain network connectivity trait (RSN-FC/SC) was chosen as the exposure and the disorder trait as the outcome. The generated *P* values were corrected using Bonferroni multiple comparison method (0.05/64 = 7.81 x10^−4^).

## Results

The GWIS analysis was first conducted to separate shared and disorder-unique genetic components, accounting for shared genetic liability. The annotation findings from two GWIS-derived genetic components of BD and SCZ are displayed in the Supplementary Tables 2–10, including SynGO and Multi-marker Analysis of GenoMic Annotation (MAGMA) annotation results. The top significant gene set in MAGMA gene-set analysis for both BDuniq and SCZuniq was the set named ‘Gene Ontology (GO): membrane protein complex’. Furthermore, for BDuniq, 14 of 221 fine-mapped genes were mapped to SynGO-annotated genes, while, 30 of 552 fine-mapped genes of SCZ_uniq_ GWAS were found to be associated with synaptic signaling and synaptic function, in the Supplementary Tables 7 and 8. Moreover, the tissue enrichment analysis using MAGMA identified the most significant associated brain region for BDuniq to be the hippocampus ( 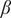 = 0·041 [0·024,0·069], *P* = 5·22 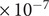 ), and the most significant one for SCZuniq to be the frontal cortex BA9 ( 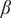 = 0·037 [0·022,0·068], *P* = 7·28 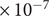 ), detailed in the Supplementary Tables 9 and 10.

**Figure 2.**
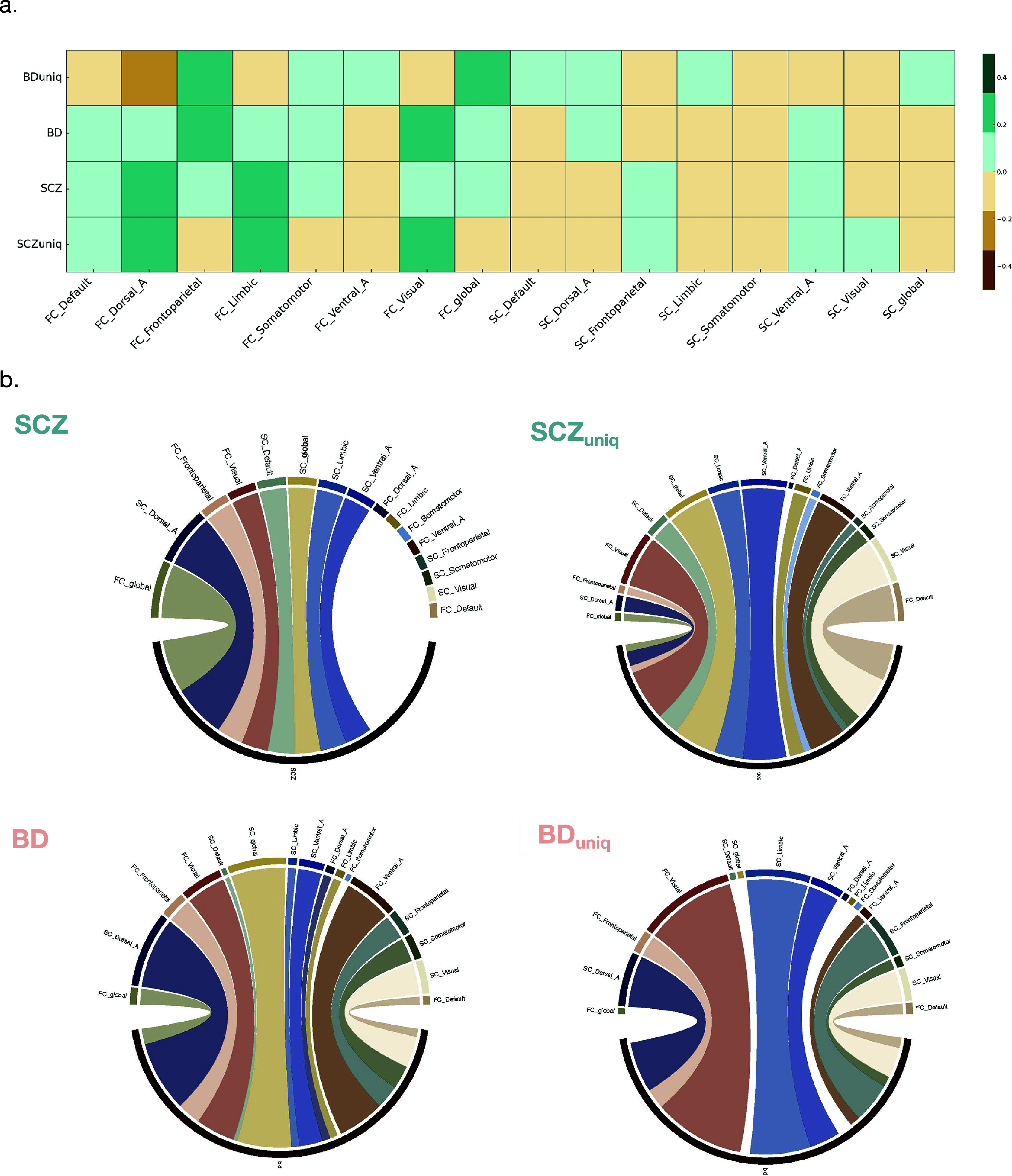
Genetic association analysis between disease traits and RSN-FC/SC. *Note*: (a) Heatmap showing genome-wide genetic association between disease traits (BD, BD_uniq_, SCZ, SCZ_uniq_) and 16 RSN-FC/SC, positive and negative correlations are indicated by the color gradient; (b) The number of loci with significant genetic assocation between disease traits (BD, BD_uniq_, SCZ, SCZ_uniq_) and 16 RSN-FC/SC. Each segment represents an RSN network or global connectivity measure, and the width corresponds to the number of loci reaching statistical significance BD; SCZ; BD_uniq_; SCZ_uniq_; RSN-FC/SC; FC/SC within DMN, FC/SC_Default; FC/SC within VAN, FC/SC_Ventral_A; FC/SC within DAN, FC/SC Dorsal_A; FC/SC within VN, FC/SC_Visual; FC/SC within LN, FC/SC_Limbic; FC/SC within SMN, FC/SC_Somatomotor; FC/SC within FPCN, FC/SC_Frontoparietal.

The global genetic correlation analyses between two disorder phenotypes and 16 neural FC and SC within RSNs indicated a positive r_g_ between SCZ and FC_DAN (r_g_ = 0.180 [0.059, 0.534], *P* = 0.0035), whereas SC_VN was the one correlated with BD with a negative coefficient (r_g_ = −0.161 [−0.269, −0.478], *P* = 0.0032). After excluding the shared genetic liability using GWIS, the correlation between SCZ_uniq_ and FC_DAN remained with a smaller magnitude of significance (r_g_ = 0.231 [0.072, 0.683], *P* = 0.0043), whereas the correlation between SCZ_uniq_ and SC_DAN became more significant (r_g_ = −0.118 [−0.219, −0.349], *P* = 0.02). Meanwhile, the correlation between BD_uniq_ and SC_VN remained less significant without the shared genetic liability with SCZ (r_g_ = −0.152 [−0.270, −0.450], *P* = 0.011), and its correlation with the SC_DAN signaled a hint of significance (r_g_ = 0.114 [0.001,0.338], *P* = 0.049). The correlation heatmap is displayed as Figure 2a, with detailed results shown in the Supplementary Table 11.

We then carried out an investigation of local correlation patterns using LAVA (Werme, van der Sluis, Posthuma, & de Leeuw, 2022). Accounting for the shared genetic liability using GWIS, the bivariate local r_g_ between SCZ/SCZ_uniq_, BD/BD_uniq_ and 16 neuroimaging traits identified 204 loci showing Bonferroni-corrected significance, shown in the Figure 2b and the Supplementary Table 12. For BD, the most significant signal arose from the locus chr3:71223282–72334704 with SC_LN, generating a *P* value of 1.64E-08 (r^2^ = 1); such signal receded with BD_uniq_ (*P* = 1.29 x10^−4^) while the locus chr14:99474534–100786189 generated the strongest signal between BD_uniq_ and SC_VN (r_g_ = 1, *P* = 9.47 x10^−7^). Intriguingly, the most significant locus for the local r_g_ with SCZ was chr11:114742318–116247377 for SC_LN (r_g_ = 1, *P* = 8.28 x10^−8^) while the local r_g_ in chr5:174662886–176180301 with FC_VN showed the strongest signal for SCZ_uniq_ (r2 = 0.622333, *P* = 1.34 x10^−7^). As for the number of significant loci, as detailed in the Supplementary Table 13, SCZ shared the most significant numbers with SC_VN (n = 13) whereas SCZ_uniq_ shared the most with SC_global (n = 6), SC_VAN (n = 6), and SC_VN (n = 6). As for BD, the highest numbers of local r_g_ were shared with SC_DAN (n = 9); after excluding the common genetic liability with SCZ, BD_uniq_ shared the most significant loci with FC_VN (n = 9).

**Figure 3.**
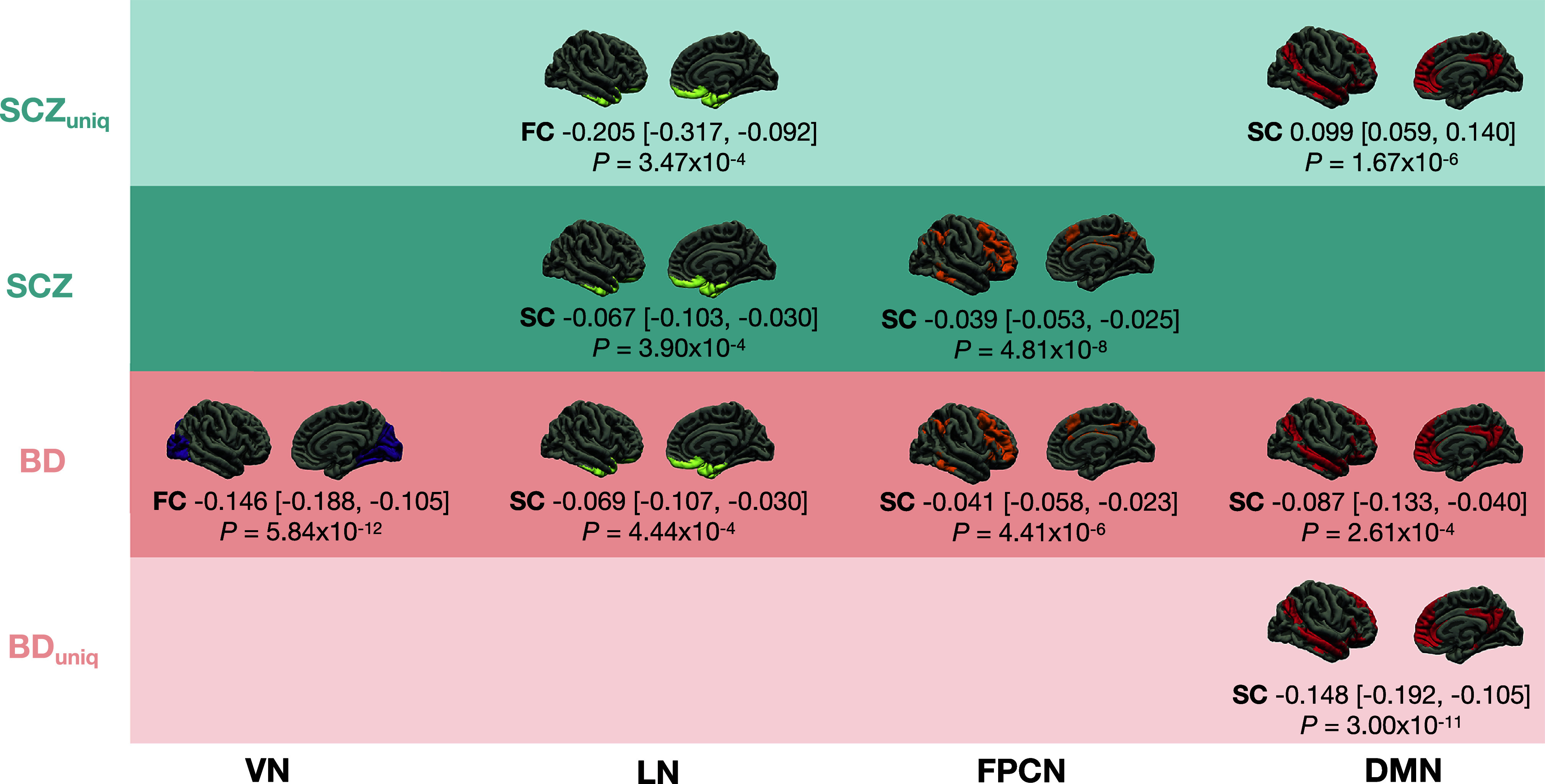
OMR results between disease traits and RSN-FC/SC. *Note*: Significant results of OMR between SCZ, BD, their GWIS-derived unique components (SCZ_uniq_ and BD_uniq_), RSN-FC/SC are shown for the significantly associated networks. Effect sizes and 95% confidence intervals are displayed for each phenotype–network pair, together with the corresponding *P* values. VN: Visual Network, LN: FPCN: FrontoParietal Control Network, DMN: Default Mode Network, FC: Fucntional connectivity, SC: Structural Connectivity.

The OMR using the omnigenic model as IVs was applied from 16 RSN-FC/SC to four disorder traits (BD, BD_uniq_, SCZ, SCZ_uniq_). The results were summarized in Figure 3 (also seen in the Supplementary Table 14). A shared overlap was detected for SC_LN and both disorders (for BD: 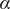 = −0.0687 [−0.107, −0.0304], *P* = 4·43E-04; for SCZ: 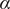 = −0.0666 [−0.103, −0.0298], *P* = 3.90 x10^−4^), and also for FPCN (for BD: 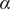 = −0.0405 [−0.0579, −0.0232], *P* = 4.41 x10^−6^; for SCZ: 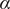 = −0.0393 [−0.0534, −0.0252], *P* = 4.81 x10^−8^). Additionally, FC_VN was found significantly associated with BD ( 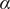 = −0.146 [−0.188, −0.105], *P* = 5.84 x10^−12^). Accounting for shared genetic liability, a significant causality was detected between SC_DMN and both BD_uniq_ and SCZ_uniq_, albeit with the opposite directions (for BD_uniq_: 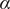 = −0.148 [−0.192, −0.105], *P* = 3.0 x10^−11^; for SCZ_uniq_: 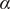 = 0.0994 [0.0587, 0.14], *P =* −1.67 x10^−6^). Besides, FC_LN ( 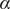 = −0·205 [−0·317, −0·0925], *P* = 3.47 x10^−4^) was causally associated with SCZ_uniq_. All the results remained statistically significant after Bonferroni corrections (0.05/64 = 7.81 x10^−4^).

## Discussion

In the current study, we endeavored to detangle the shared and unique genetic architecture of BD and SCZ and their relationships with brain network connectivity alterations, specifically, connectivity within RSNs. Using the advanced mathematical algorithm, we generated new GWAS results for BD and SCZ, presumably eliminating their common genetic liability, only leaving the disorder-unique genetic components (BD_uniq_ and SCZ_uniq_). Further, we investigated their genetic relationships, both non-directional and directional, RSN connectivity alterations.

Many lines of evidence point to a remarkable overlap between BD and SCZ (Girgenti et al. (2012)). How to carve up the biological delineator for each disorder plays a pivotal role in understanding and improving the current nosology and treatment. While it remains enormously challenging to collect unique samples based on current phenomenology-oriented diagnosis criteria, utilizing genome-wide information gleaned from GWASs and mathematical algorithms could shed promising light on the shared and unique genetic underpinning of two disorders (Dang et al. (2023)). Programs such as MiXeR (Frei et al., 2019) quantify the number of causal variants shared by two phenotypes, but they fall short of providing detailed knowledge regarding these causal variants. The GWIS, on the other hand, applies functions to well-powered GWAS summary statistics and derives the secondary results. One of the main advantages of GWIS is that its results enable downstream analyses such as the calculation of genetic correlation and MR study, which take GWAS summary statistics as input. It is important to clarify that the GWIS-derived ‘unique’ components do not represent fully independent biological mechanisms. Rather, they capture both the residual genetic signal and the potential disorder-level differences that become apparent after the shared variance between SCZ and BD.

For GWIS-derived unique components, subsequent fine-mapping and tissue enrichment analyses revealed that both disorders are significantly enriched in the GO term membrane protein complex and the SynGO term synapse, consistent with prior evidence implicating synaptic and membrane-related dysfunction in both disorders (Liu et al. (2023)). There is a long history of hypotheses and emerging evidence concerning synapses and receptors on neuron membranes in BD and SCZ (Verschueren et al. (2020)). The SynGO database further underscores the critical role of synapse in their neuropathology. Although the two GWIS-derived unique components show overlapping pathway enrichments, these shared signals do not necessarily indicate identical biological mechanisms. Further work will be needed to clarify whether these overlaps reflect true convergence or arise from residual shared architecture. Moreover, the majority of brain tissues expressions were found to be associated with BD_uniq_ and SCZ_uniq_. Among them, the frontal cortex and hippocampus appeared among the top-enriched by SCZ_uniq_ and BD_uniq_, respectively, based on the ranking of enrichment P values. This provides a potential mechanistic clue in brain regions or networks whose synapse or neural dysfunctions may contribute to each condition. This suggests that apparently overlapping biological themes may still involve disorder-specific molecular mechanisms. Another explanation is that genetic heterogeneity underlies clinical heterogeneity, rather than dichotomous diagnostic heterogeneity.

Indeed, the subsequent global and local genetic correlation analyses corroborate our preliminary theory that different network connectivity is associated with each different condition. For example, the global genetic correlation analyses suggested the association of attention networks with both disorders, but the pattern of associations differed between them. Introduced by Corbetta and Shulman, attention networks are the frontoparietal systems which are activated when perceiving different kinds of objects in the surroundings (Corbetta & Shulman, 2011). Attention deficits have been identified by different studies as the key features of both SCZ and BD, especially of their psychotic symptoms. Larsson *et al.* carried out a co-segregating study of SCZ, BD and attention-deficit hyperactivity disorder (ADHD) and found an increased risk of predisposing to BD and SCZ for the first-degree relatives of ADHD probands (Larsson et al., 2013). Our findings suggest that the DAN may play an important role, as SC_DAN showed relatively higher number of significant associated loci across BD (N = 9), SCZ (N = 6), BD_uniq_ (N = 5), and SCZ_uniq_ (N = 2). Although altered attention network activities have been reported in both disorders, which may have been influenced by overlapping symptoms and clinical heterogeneity. The findings from the current study, by excluding genetic covariation between SCZ and BD, provided additional evidence that DAN may contribute to each disorder through partly overlapping yet distinct pathways.

Our study also detected a comprehensive association analysis at both the genome-wide and local levels, revealing a complex interplay of positive and negative genetic effects. Given the intrinsic genetic landscape and the ongoing debate around the dysconnectivity hypothesis, we utilized OMR to infer potential causal relationships from brain connectivity to the original and unique parts of BD and SCZ. Of note, both SCZ and BD showed negative association with the SC_LN. After excluding the shared genetic disposition, SCZ_uniq_ continued to show a stronger negative association with FC_LN, whereas BD_uniq_ did not retain a similar pattern. It suggests that limbic-related genetic components may remain more specifically linked to SCZ than to BD. And LN is responsible for not only behavioral and emotional responses but also dopaminergic projections, contributing to neurophysiology and treatments of two disorders (Bi, Che, & Bai, 2022; Dugre, Bitar, Dumais, & Potvin, 2019; Wang et al., 2022). The limbic dysfunction has long been recognized as *mea culpa* of both SCZ and BD, especially their psychosis symptoms, which may partially align with our findings.

The frontoparietal network plays an important role in cognitive control and working memory, whose disruptions were found to be associated with core symptoms in both disorders (Baker et al., 2014; Dong et al., 2018; Ye et al., 2023). In our analysis, both SCZ and BD showed negative associations with SC_FPCN, but these associations did not remain significant after accounting for shared genetic liability in SCZ_uniq_ and BD_uniq_. This pattern suggests that reduced frontoparietal connectivity may reflect a general cognitive dysfunction shared by the two disorders. Meanwhile, the SC within DMN showed opposite effects on BD_uniq_ and SCZ_uniq_. A prior structural MRI study showed that overall reductions in SC in both disorders, but SCZ has additionally been associated with increased fractional anisotropy in certain fiber tracts within the default network (Ji et al., 2019). Other studies have found that SCZ patients displayed more impairments in SC than BD patients (Cea-Canas et al., 2019; Repple et al., 2023). These heterogeneous findings may reflect differences in clinical state, symptom profiles, or medication effects, making it plausible that the GWIS-derived unique components capture disorder-level opposite effect in DMN involvement once the shared signal is removed.

Both SCZ and BD have been found to be associated with visual perception impairment (Adámek, Langová, & Horáček, 2022; Fernandes, Silverstein, Almeida, & Santos, 2019). In line with this, our LAVA results showed that the VN exhibited one of the largest numbers of loci with significant local genetic correlation across SCZ, BD, BD_uniq_, and SCZ_uniq_. The visual impairments in both disorders have been reported in the same direction, for example, the higher visual discrimination thresholds (Løchen et al., 2023), poorer visual acuity (Shoham et al., 2023), and faster bi-stable switch (Killebrew et al., 2024), compared to healthy controls. However, in the OMR analysis, only BD showed a significant association with VN connectivity. This discrepancy is expected. Although we describe the assumptions of the OMR framework, the results should still be interpreted cautiously. Therefore, our findings from genetic correlation and MR analysis provide suggestive evidence of possible directional effects but cannot establish definitive causal claims. Longitudinal individual level data will help refine these interpretations.

Although promising and guaranteeing further explorations, our study has a few limitations. First, the available brain connectivity GWAS are based on a modest sample size and lack an independent replication cohort, which may increase uncertainty in effect size estimates and the risk of false-positive associations. Our analyses were based on European-ancestry GWAS, which may limit generalizability to other populations. As more diverse imaging GWAS become available, future work can evaluate whether these network-genetic relationships hold across ancestries. Additionally, neuroimaging preprocessing needs standard and universal imaging preprocessing methods. Ongoing international efforts to harmonize MRI preprocessing across sites will likely improve the consistency and robustness of imaging-genetic associations. Last but not least, more dimensional and precise phenotype should be incorporated in the following genetic study to model clinical heterogeneity and comorbidity (Waszczuk et al., 2023).

In summary, our findings separate the shared and disorder-unique genetic components of SCZ and BD and demonstrate how these components relate to patterns of brain network connectivity. The integrated genetic and network-level evidence provides a clearer view of the common and distinct biological components involved. Although further replication and individual-level imaging–genetic data will be needed, this framework may eventually support early diagnostic stratification and guide the development of more targeted interventions, including neurostimulation approaches that focus on altered connectivity rather than DSM categories.

## Supporting information

10.1017/S0033291726104413.sm001 Ren et al. supplementary material Ren et al. supplementary material

## Data Availability

All the GWAS summary statistics used in this study are publicly available. The SCZ and BD GWAS data are accessible on the Psychiatric Genomics Consortium website. The imaging GWAS summary statistics from Tissink et al. (2023) are available through the corresponding repository (link provided in their publication).

## Supplementary material

The supplementary material for this article can be found at http://doi.org/10.1017/S0033291726104413.
